# Prognostic role of stereotactic body radiation therapy for elderly patients with advanced and medically inoperable pancreatic cancer

**DOI:** 10.1002/cam4.1164

**Published:** 2017-08-23

**Authors:** Xiaofei Zhu, Fuqi Li, Xiaoping Ju, Fei Cao, Yangsen Cao, Fang Fang, Shuiwang Qing, Yuxin Shen, Zhen Jia, Huojun Zhang

**Affiliations:** ^1^ Changhai Hospital Affiliated to Second Military Medical University Shanghai China

**Keywords:** Cyberknife, elderly, pancreatic cancer, stereotactic body radiation therapy, survival

## Abstract

The role of stereotactic body radiation therapy for the elderly with advanced or medically inoperable pancreatic cancer was still debated. Therefore, we evaluated the value of stereotactic body radiation therapy and its association with survival of those patients. A total of 417 elderly patients were retrospectively reviewed from 2012 to 2015. Overall survival (OS), progression‐free survival (PFS), local recurrence‐free survival (LRFS), distant metastasis‐free survival (DMFS), and toxicities were analyzed. Prescription doses ranged from 30–46.8 Gy in 5–8 fractions. Median age was 73 years old. Median OS, PFS, LRFS, and DMFS were 10, 8, 10, and 9.5 months, respectively. One‐year OS, PFS, LRFS, and DMFS rate were 35.5%, 18.2%, 26.6%, and 27.1%, respectively. Tumor stage and tumor response at 6 months and CA19‐9 levels normalization at 3 months after treatment were independent predictors of OS, PFS, LRFS, and DMFS. Patients with early‐stage cancer, better tumor response, and normalization of CA19‐9 levels had significantly longer OS, PFS, LRFS, and DMFS. Patients with the prodrug of 5‐FU and radiotherapy had longer survival than those with gemcitabine‐based chemotherapy and radiotherapy. Patients who received BED_10_ ≥ 60 Gy achieved better tumor response compared with those who received BED_10_ < 60 Gy. Two patients had grade 4 intestinal strictures. No grade 3 or higher hematologic toxicities occurred. Stereotactic body radiation therapy is safe and effective for elderly patients with advanced or medically inoperable pancreatic cancer. Early efficacy could be predictive of prognosis. Higher doses may be associated with efficacy but need further investigation.

## Introduction

Despite advances in multidisciplinary managements, pancreatic cancer remains a highly lethal disease. It is the fourth leading cause of cancer mortality among both men and women in the United States 1, with a surprisingly low 5‐year survival rate of 7% and increasing incidence and death rates within these 3 years 1, which was also similar in China 2. Although surgical resection may be the only possibility for cure, only 15–20% of the patients are candidates for curative resection at presentation 3, 4 due to its insidious character in the locally advanced stage and unsuccessful population‐based screenings. The overall 5‐year survival rate of those even with *R*_0_ resection with or without adjuvant therapy is less than 20% 5, 6, 7, 8, 9. Moreover, pancreatic cancer is the fourth and fifth leading cause of cancer death in men aged 60–79 years and over 80 years, respectively; while ranking fourth in women both aged 60–79 years and over 80 years in the United States 1.

It has been concluded that surgery for pancreatic adenocarcinoma is not contraindicated in the elderly with careful selection of those deemed operable 10, 11. Therefore, radiation or chemotherapy may play a more important role in the treatment of advanced or medically inoperable pancreatic cancer. However, multimodality treatment for elderly patients remains controversial because no consensus has been reached on the optimal treatment. Although it has been shown that adjuvant chemoradiotherapy is associated with increased survival but this effect is limited 12, 13. Despite the conformal methods of treatment delivery of conventional radiotherapy, significant acute and late side effects may develop. Besides, prolonged conventional radiotherapy may delay delivery of systemic doses of chemotherapy.

Given the shortcomings of conventional radiotherapy, stereotactic body radiation therapy (SBRT) is a promising option due to its precise treatment delivery with a sharp dose fall off outside the target area and acceptable toxicity, especially for the elderly patients with advanced and medically inoperable pancreatic cancer. Also, the shorter duration of SBRT compared to conventional fractionation can be advantageous among patients with short life expectancy. In this study, the safety and efficacy of SBRT in elderly patients with advanced and medically inoperable pancreatic cancer were explored.

## Methods

### Patients and pretreatment assessment

Patients aged over 65 years with advanced or medically inoperable pancreatic cancer treated with SBRT were candidates in this study. Written informed consent was required prior to treatment. Moreover, laboratory tests or imaging studies were requested for their medical evaluations.

### Eligibility

Histopathologic examinations were requested for all patients with clinical suspicion of pancreatic cancer based on imaging studies. Further dedicated imaging of the pancreas, including enhanced MRI for identifying minor metastasis in the liver not confirmed in CT and details of tumor local invasion, if deemed necessary, would be performed before any study‐related procedures. In our study, most patients were diagnosed with locally advanced pancreatic cancer. For patients with metastatic pancreatic cancer, SBRT was performed for palliative purposes. In addition, the cohort included patients inoperable due to comorbidities regardless of the stage of the pancreatic cancer. All definitions are based on the NCCN guidelines 14.

#### Inclusion criteria

Age of 65 years or more; ECOG performance status (ECOG PS) ≤2; normal renal function (serum creatinine ≤ 2.0 mg/dL); normal liver function (serum total bilirubin ≤ 3.0 mg/dL, serum AST ≤ 2.5 of the upper limit of normal, serum ALT ≤ 2.5 of the upper limit of normal); routine blood test: WBC ≥ 3.5 × 10^9^/L, neutrophils ≥ 1.5 × 10^9^/L, hemoglobin ≥ 80 g/L, and platelet ≥ 70×10^9^/L.

#### Exclusion criteria

Ampulla of Vater cancer; patients under the age of 65; ECOG PS >2; gastrointestinal inflammation or other diseases (especially active inflammatory bowel, nonhealing peptic ulcer, gastrointestinal bleeding, or perforation within 6 months); Impaired organ functions: (1) Heart failure (NYHA III‐IV); (2) Respiratory failure; (3) Renal insufficiency (serum creatinine >2.0 mg/dL); (4) Hepatic insufficiency (serum total bilirubin > 3.0 mg/dL, serum AST > 2.5 of the upper limit of normal, serum ALT > 2.5 of the upper limit of normal or Child‐Pugh class B or C); (5) Routine blood test: WBC < 3.5 × 10^9^/L, neutrophils < 1.5 × 10^9^/L, hemoglobin < 80 g/L, platelet < 70 × 10^9^/L, or other hematological diseases; and (6) Severe nervous system diseases; patients who were not willing to comply with subsequent treatment plans, tests, and other study procedures.

Due to better diagnostic yield, fine‐needle aspiration was preferred for all patients suspected of pancreatic cancer. However, patients with high risk of bleeding, pancreatitis, pancreatic fistula, or intolerance of biopsies were not recommended to receive biopsies. Therefore, it was crucial and mandatory to establish the clinical diagnosis of pancreatic cancer with caution by the multidisciplinary team based on medical history and all available tests before treatment.

### Therapeutic interventions

#### SBRT

The protocol was based on our previous publication 15. SBRT was delivered via CyberKnife^®^ (Accuray Incorporated, Sunnyvale, CA), an image‐guided frameless stereotactic robotic radiosurgery system. Prior to the treatment, a plain CT and a contrast‐enhanced pancreatic parenchymal CT were performed for radiation treatment planning and target delineation. Pretreatment diagnostic imaging was coregistered to the simulation CT in cases in which the patient was unable to tolerate intravenous contrast. Gross tumor volume (GTV) was delineated as a radiographically evident gross disease by contrast CT acquired from the portal‐venous phase. At the discretion of the physician, clinical target volume (CTV) encompassing areas of the potential subclinical disease spread was also designated. In most cases, the CTV equaled GTV. A 2–5 mm expansion margin was included to determine the planning target volume (PTV). When the tumor was adjacent to critical organs, the expansion of PTV outside of CTV in this direction should be avoided. Therefore, the margin expansion was allowed to be nonuniform. At least ninety percent of PTV should be covered by the prescription dose. The prescription isodose line was limited to 70–80%, which would restrict the tumor *D*_max_. The prescribed doses were based on tumor geometry and location. In particular, these doses would be reduced if the tumor was approximately one third or more of the duodenum or stomach circumference, or if the tumor abutted the bowel in only one area, as determined by the relationship of the tumor to the duodenum in axial, coronal, and sagittal planes in CT scans, or the space between the tumor and the bowel wall was <3 mm. Normal tissue constraints were according to the American Association of Physicists in Medicine guidelines in TG‐101 16. The formula of biological effective dose (BED) was as follows: $\text{BED}\;=\;\text{nd}\;\times \;\left(1\;+\;\frac{d}{\alpha /\beta }\right),\;\frac{\alpha }{\beta }\;=\;10$ (BED_10_).

### Outcomes

The primary endpoint of the study was overall survival. Additionally, data on other endpoints for each patient were collected, including progression‐free survival (PFS), local recurrence‐free survival (LRFS), distant metastasis‐free survival (DMFS), and radiation‐induced acute and late toxicities. PFS, LC, and DMFS were all associated with treatment response, as determined by the RECIST criteria (version 1.1). Radiation‐induced acute toxicities within 90 days after treatment were determined by the Radiation Therapy Oncology Group, “Acute radiation morbidity scoring criteria”. Late toxicities occurring 3 months after SBRT were evaluated by the Radiation Therapy Oncology Group/European Organization for Research on the Treatment of Cancer, “Late radiation morbidity scoring schema”. The adverse effects of chemotherapy were assessed by Common Terminology Criteria for Adverse Effects (CTCAE) v4.03.

### Statistical analysis

Statistical testing was performed using SPSS version 19.0 (IBM Corp, Armonk, NY). Patient characteristics and demographic data were summarized by descriptive statistics. Patients lost to the consecutive follow‐ups were censored at the last follow‐up. Median survival was calculated with a confidence interval (CI) of 95%. OS, PFS, LRFS, and DMFS were calculated via the Kaplan–Meier method. Univariate Cox regression analysis was employed to test for associations between potential prognostic factors and survival. Factors associated with survival on univariate analysis were taken as covariates in a multivariate proportional hazards regression model for survival. Two‐sided *P* values <0.05 were considered statistically significant.

## Results

### Patient characteristics

A total of 420 patients with at least 65 years of age were treated in our hospital starting in 2012. Due to three patients lost to follow up, 417 elderly patients were enrolled in the study between January 2012 and December 2015. Demographic, clinical, and treatment characteristics were listed in details in Table 1. Median follow‐up was 11 months (range, 4–28 months). Median age at diagnosis was 73 years (range, 65–90 years). Prescription dose varied from 30–46.8 Gy delivered in five to eight fractions.

**Table 1:** Patient characteristics

Characteristics	Value
All patients	417
Gender	
Male	257
Female	160
Age (years)	73 (65–90)
ECOG PS	
0	183 (43.9%)
1	209 (50.1%)
2	25 (6.0%)
Stage	
Borderline resectable	105 (25.2%)
Locally advanced	218 (52.3%)
Metastatic	94 (22.5%)
Medically inoperable	291 (69.8%)
Poor physical condition	51 (12.2%)
Too old to be operated	117 (28.6%)
Declining surgery	123 (29.5%)
Tumor locations	
Head	276 (66.2%)
Body and tail	141 (33.8%)
Tumor diameter (cm)	3.6 (1–8.4)
Baseline CA19‐9 (U/mL)	
≤30	71 (17.0%)
30–100	62 (14.9%)
>100	284 (68.1%)
Previous treatment	
Surgery alone	39 (9.4%)
Chemotherapy alone	87 (20.9%)
Surgery and chemotherapy	14 (3.4%)
Other treatment	33 (7.9%)
Treatment naïve	244 (58.4%)
Prescription dose	30–46.8 Gy/5–8f
BED_10_	61.92 Gy (range, 48–94.08 Gy)

### Response rate and survival

All patients completed the SBRT treatment and were evaluable for tumor response, which was determined by RECIST criteria 6 months after treatment. Normalization of CA19‐9 levels 3 months after radiotherapy was also included as a response to the treatment for those with baseline CA19‐9 levels above upper limit of normal. Twenty‐one patients had complete response, 95 had partial response, and 264 had stable disease. Of the 346 patients with high baseline CA19‐9 levels, 79 returned to normal levels (Table 2). Among 116 patients with CR or PR, 54 had normalization of CA19‐9 3 months after SBRT, while 41 remained normal levels of CA19‐9 and 21 still with over upper limit of normal.

**Table 2:** Responses to treatment

Responses	Number (%)
Tumor response	
CR	21 (5.1%)
PR	95 (22.8%)
SD	264 (63.3%)
PD	37 (8.8%)
CA19‐9 level after radiation	
Decline to normal level	79 (18.9%)
Remain normal	71 (17.0%)
Over upper limit of normal	267 (64.1%)

**Figure 1:**
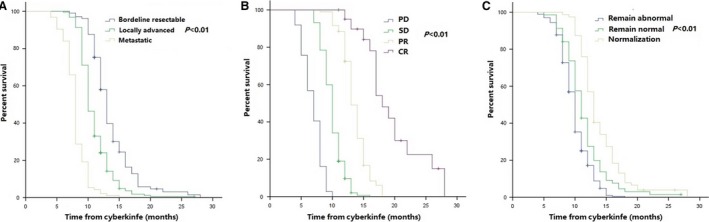
OS of patients with different stages (A), different tumor response (B), and different changes in CA19‐9 levels (C) after treatment.

Median OS of all patients was 10.0 months (95% CI 9.7–10.3 months). One‐year OS rate was 35.5% (95% CI 32.5%–41.9%) (Fig. S1A). Multivariate analysis indicated that stage (*P* < 0.001; hazard ratio [HR] [95% CI], 2.38 [1.98–2.87]), tumor response (*P* < 0.001; HR [95% CI], 0.20 [0.16–0.25]), and normalization of CA19‐9 (*P* = 0.001; HR [95% CI], 0.76 [0.65–0.88]) were associated with OS (Table S1). Median OS and comparisons of OS of patients at different stages, with different responses, and different CA19‐9 levels were detailed in Table S2 and Figure 1.

**Figure 2:**
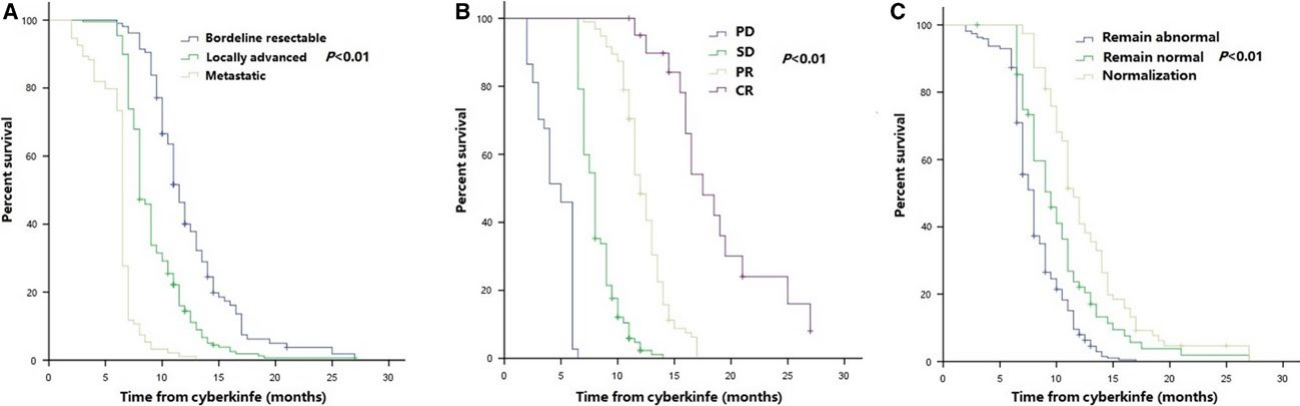
PFS of patients at different stages of disease (A), different tumor response (B), and different changes in CA19‐9 levels (C) after treatment.

Median PFS among all patients was 8.0 months (95% CI 7.6–8.3 months). Median PFS at 1 year was 18.2% (95% CI 14.2%–21.6%) (Figure S1B). Stage (*P* < 0.001; HR [95% CI], 3.06 [2.53–3.72]), tumor response (*P* < 0.001; HR [95% CI], 0.09 [0.07–0.12]), and normalization of CA19‐9 (*P* = 0.001; HR [95% CI], 0.72 [0.62–0.83]) were correlated with PFS in multivariable analysis (Table S3). Additionally, further analysis of PFS in the above three groups was presented in Figure 2 and Table S2.

### Local control and distant metastasis‐free survival

**Figure 3:**
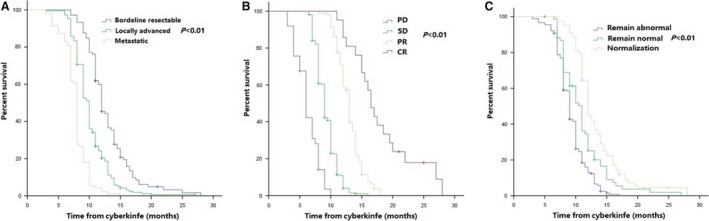
LRFS of patients with different stages (A), different tumor response (B), and different changes in CA19‐9 levels (C) after treatment.

Median local recurrence‐free survival (LRFS) in all patients was 10.0 months (95% CI 9.6–10.3 months). LRFS at 1 year was 26.6% (95% CI 24.4%–33.0%) (Figure S2A). Patients with early stage of cancer (*P *< 0.001; HR [95% CI], 1.81 [1.52–2.15]), good response (*P *< 0.001; HR [95% CI], 0.23 [0.19–0.28]), and normalization of CA19‐9 (*P *= 0.001; HR [95% CI], 0.76 [0.66–0.89]) after irradiation had higher probability of LRFS according to our multivariate analysis (Table S4). Comparisons of LRFS among different stages, tumor response, and CA19‐9 levels were shown in Figure 3 and Table S5.

**Figure 4:**
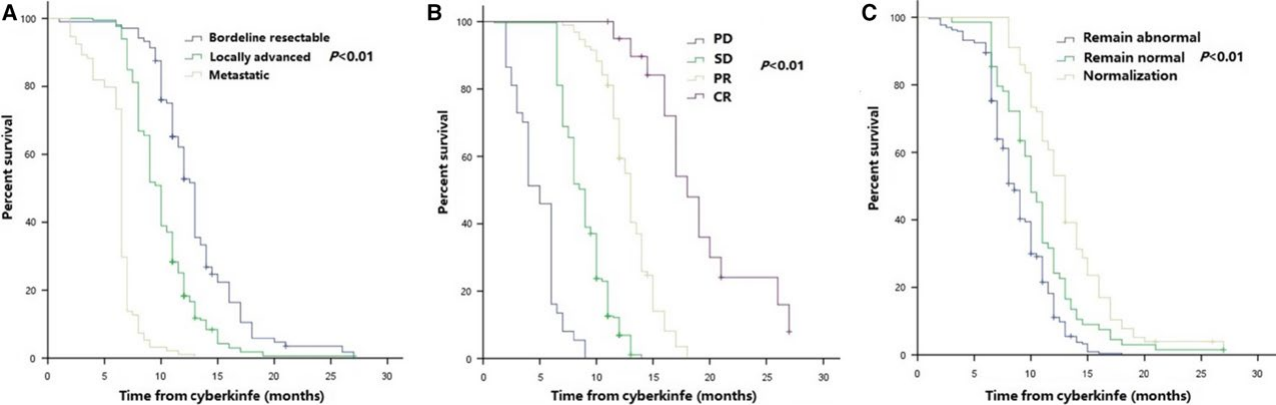
DMFS of patients with different stages (A), different tumor response (B), and different changes in CA19‐9 levels (C) after treatment.

Median distant metastasis‐free survival (DMFS) in all patients was 9.5 months (95% CI 9.1–9.9 months). The 1‐year DMFS rate was 27.1% (95% CI 24.6%–33.2%) (Figure S2B). On multivariate analysis, stage (*P *< 0.001; HR [95% CI], 1.81 [1.52–2.15]), tumor response (*P *< 0.001; HR [95% CI], 0.23 [0.19–0.28]), and normalization of CA19‐9 (*P *= 0.001; HR [95% CI], 0.76 [0.66–0.89]) remained as independent predictors of DMFS (Table S6). Furthermore, patients with early stage of cancer, good tumor response, and normalization of CA19‐9 levels tended to have better DMFS (Figure 4 and Table S5).

The mode of initial disease progression was local recurrence in 163 patients (15.1%), distant metastasis in 222 patients (53.2%), and local and distant failure in 17 patients (4.1%). Chemotherapy was performed for 47 patients while 11 patients received re‐irradiation. Among patients with re‐irradiation, 4 patients had local progression with a median prescription dose of 30–40 Gy/6–8f. Four and three patients with liver and lung metastasis were re‐irradiated with a median prescription dose of 40–45 Gy/5f and 40–50 Gy/5–6f.

### Outcomes of different treatment

Forty‐seven patients received gemcitabine‐based chemotherapy before radiotherapy while S‐1 (a prodrug of 5‐fluorouracil comprising of tegafur, gimeracil, and oteracil) was given as the initial treatment for 40 patients. Upfront surgery was performed in 32 patients. There were significant differences between patients treated with gemcitabine‐based chemotherapy + SBRT and those receiving S‐1 + SBRT regarding OS, PFS, LRFS, and DMFS. The OS of patients with surgery + SBRT, gemcitabine‐based chemotherapy + SBRT, and S‐1 + SBRT were 9.0 months (95% CI 8.4–9.7 months), 9.0 months (95% CI 8.3–9.7 months), and 10.0 months (95% CI 8.9–11.0 months) (the latter two group: *P* = 0.009), respectively. The PFS of the three groups was 7.5 months (95% CI 7.0–8.0 months), 7.0 months (95% CI 6.5–7.4 months), and 7.0 months (95% CI 6.6–7.4 months) (the latter two group: *P* = 0.015), respectively. The LRFS of those three groups was 9.0 months (95% CI 8.0–10.0 months), 8.0 months (95% CI 7.7–8.2 months), and 9.0 months (95% CI 8.6–9.4 months) (the latter two group: *P* = 0.012), respectively. The DMFS of the three groups was 8.0 months (95% CI 7.6–8.3 months), 8.0 months (95% CI 6.7–9.3 months), and 9.3 months (95% CI 7.8–10.8 months) (the latter two group: *P* = 0.001), respectively.

### Doses and other outcomes

Median BED_10_ was 61.92 Gy (range, 48–94.08 Gy). BED_10_ < 60 Gy and BED_10_ ≥ 60 Gy were further analyzed to evaluate their association with tumor response at 6 months and normalization of CA19‐9 levels at 3 months after SBRT. It was identified that patients receiving BED_10_ ≥ 60 Gy tended to have better tumor response (HR [95% CI], 0.508 [0.11–0.91]). Additionally, after stratification according to tumor locations, high BED_10_ was correlated well with better tumor response (*R* ^2^ = 0.149, *P*<0.001). However, no correlation between BED_10_ and normalization of CA19‐9 levels was found.

### Toxicity

Toxicities were evaluated in the enrolled 417 patients. Regarding gastrointestinal toxicities, grade 4 gastrointestinal strictures were observed in two patients (0.5%). For these two patients, tumors were closely adjacent to the descending part and horizontal part of the duodenum with invasion of surrounding major vessels. They were prescribed with doses of 40.2 Gy/6f and 42 Gy/6f, respectively. Stent implantation was performed for the two patients instead of salvage surgery. No grade 3 or higher late gastrointestinal toxicities occurred. The other grade 1 or 2 nonhematologic toxicities observed were anorexia, nausea, mild diarrhea, and mild abdominal pain. All patients recovered with conservative management. In addition, grade 1 or 2 neutropenia, leukopenia, or thrombopenia was observed in 63 patients (15.1%). There were no cases showing life‐threatening toxicity, and no treatment‐related deaths occurred.

## Discussion

Our data suggested that early stage of pancreatic cancer, better tumor response, and normalization of CA19‐9 were independently predictive of better prognosis. We also observed a significant correlation between tumor response and BED_10_ ≥ 60 Gy, but prescription doses did not impact survival. Besides, similar survival was found in patients with S−1 + SBRT and surgery + SBRT, as well as longer than that of patients receiving gemcitabine + SBRT.

Although previous studies have demonstrated that SBRT combined with chemotherapy seemed to be superior to standard chemoradiotherapy with respect to toxicity and efficacy 17, 18, 19, no consensus has been reached on the optimal options, yet without detailed clinical practice of SBRT for those elderly patients with advanced and medically inoperable pancreatic cancer due to no clinical trials about treatment or SBRT for them. Nevertheless, retrospective analysis still identified SBRT as a viable modality for them in the current best practice and priorities for research in radiation oncology 20. One study has explored the outcomes of SBRT for 26 patients with age over 80 21. Ten patients received SBRT combined with chemotherapy. The median dose was 24–35 Gy while the most common fractionation schedule was 30–36 Gy in three fractions. Median OS was 7.6 months and 1‐year OS rate was 34.6%. Median local control was 11.5 months while 1‐year actuarial rate was 41.2%. Median freedom from metastatic disease was 8.4 months and 1‐year actuarial rate was 41.4%. Another study has reported their experience of SBRT for elderly patients with medically inoperable pancreatic cancer 22. Twenty patients were given the following prescription dose: 35 Gy/5f, 30 Gy/5f, or 36 Gy/3f. Median OS and recurrence‐free survival was 6.4 months and 6.8 months, respectively. In our study, the median OS, PFS, LRFS, and DMFS were 10.0, 8.0, 10.0, and 9.5 months, respectively. The overall survival was longer than that in previous studies while progression‐free survival, including local recurrence and distant metastasis‐free survival, was similar. One of the underlying reasons may be that patients enrolled in our investigation were younger, who were aged over 65. Besides, fewer patients (58.4%) were treatment naïve in our study than those (65.0%) in Kim et al. 21. However, more patients (22.5%) were diagnosed with metastatic pancreatic cancer at the initial of SBRT in our study than those in previous studies (8.0% and 0%, respectively), which may negatively impact the prognosis. Additionally, unpublished data at Johns Hopkins has revealed favorable results with SBRT for 29 patients with age over 70, reporting a median overall survival of 13.0 months, comparable to our investigation.

In these two studies, there were no acute or late grade 3+ toxicities in Kim et al. 21, while Yechieli et al. 22 reported that 7 (35%) and 3 (15%) patients had grade 1–2 and grade 3–4 toxicities. Of these three patients, two had dehydration and one with episodes of gastrointestinal bleeding. In our study, only two patients had grade 4 gastrointestinal strictures. The incidence was much lower than that in those two studies. Milder and acceptable toxicities in our patients may be attributable to lower prescription doses, if patients were deemed at a high risk of adverse effects. Furthermore, in our delivery of treatment plans, the PTV margin would not be generated at the expansion of GTV at this direction, in which the tumor closely abutted to the organs at risk, which may reduce the radiation doses to normal tissues.

Although there was no correlation between the dose and OS, PFS, LRFS, or DMFS, patients receiving BED_10_ ≥ 60 Gy achieved better tumor response 6 months after SBRT than those who received BED_10_ < 60 Gy in our study. Meanwhile, high BED_10_ was not a significant predictive factor of normalization of CA19‐9 levels after treatment. However, in our study, there was a trend toward longer survival with higher prescription doses. Similar results were also found in the study investigated by Kim et al., in which patients with prescription doses greater than 20 Gy tended to have improved local control 21. The correlation between doses and prognosis is still needed to be further investigated.

Our study is limited by its retrospective nature. Hence, potential prognostic factors need to be further assessed in prospective studies. Also, this is a nonrandomized study, thus patient selection may have influenced the outcomes and determination of the appropriate treatment variable should be based on the clinical judgment of the physician.

In conclusion, SBRT is safe and effective for elderly patients with advanced pancreatic cancer, which may be an alternative for those not eligible for surgical resection. And combination of SBRT and chemotherapy might be beneficial for elderly patients with good performance status. Stage, tumor response, and normalization of CA19‐9 levels were correlated well with prognosis while BED_10_ was the only predictor of tumor response. Further investigations need to identify optimal treatment based on SBRT for the elderly patients.

## Conflict of Interest

The authors declare no potential conflicts of interest.

## Supporting information

Figure S1. OS (A) and PFS (B) of all patients.

Figure S2. LRFS (A) and DMFS (B) of all patients.

Table S1. Factors associated with OS.
Table S2. OS and PFS of patients at different stages, different responses, and different changes in CA19‐9 levels.
Table S3. Factors associated with PFS.
Table S4. Factors associated with LRFS.
Table S5. LRFS and DMFS of patients with different stages, different responses, and different changes in CA19‐9.
Table S6. Factors associated with DMFS.
