# Supplementary figures and images for: Prognostic role of stereotactic body radiation therapy for elderly patients with advanced and medically inoperable pancreatic cancer
Source: Cancer Med. 2017 Aug 23;6(10):2263–70. doi: 10.1002/cam4.1164 (PMC5633558; doi:10.1002/cam4.1164)

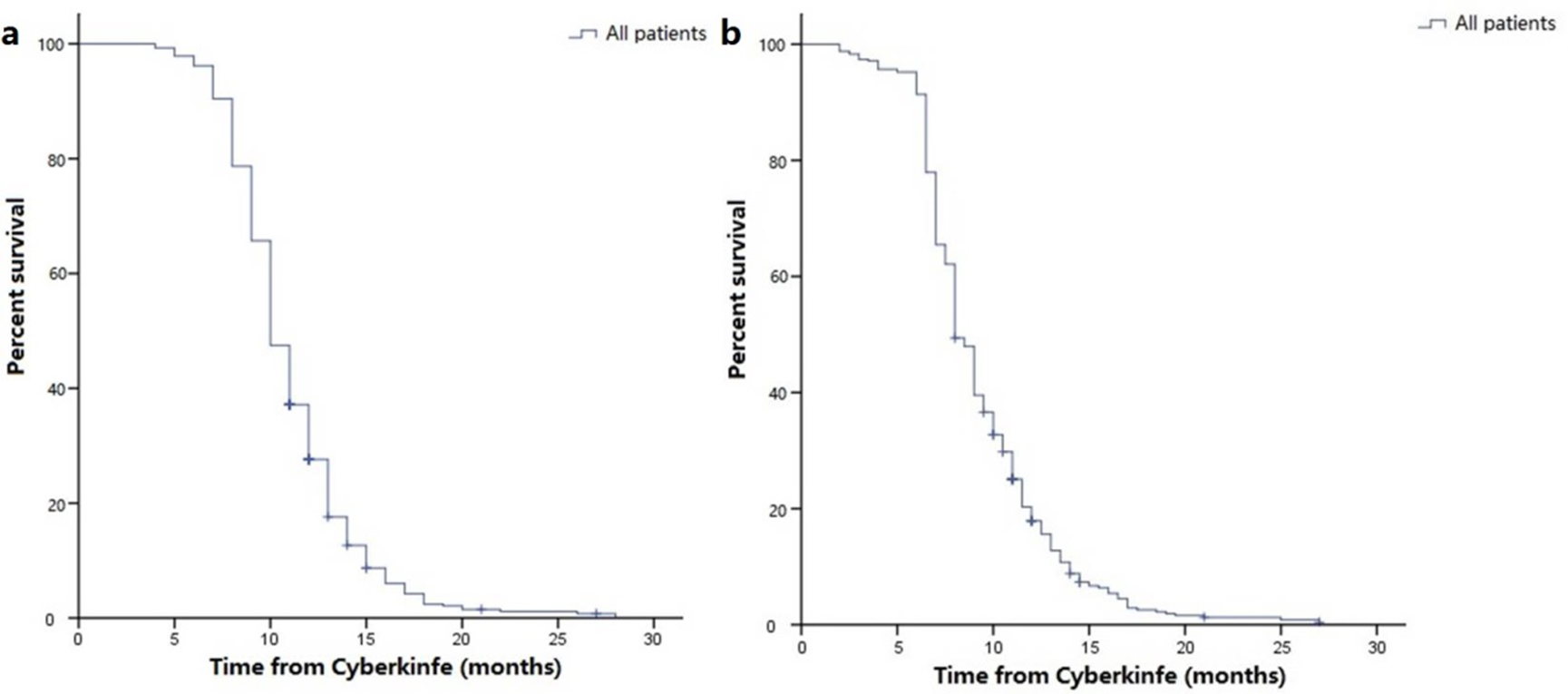

Supplement: Supplementary file 1 — Figure S1. OS (A) and PFS (B) of all patients. [file CAM4-6-2263-s001.tif]

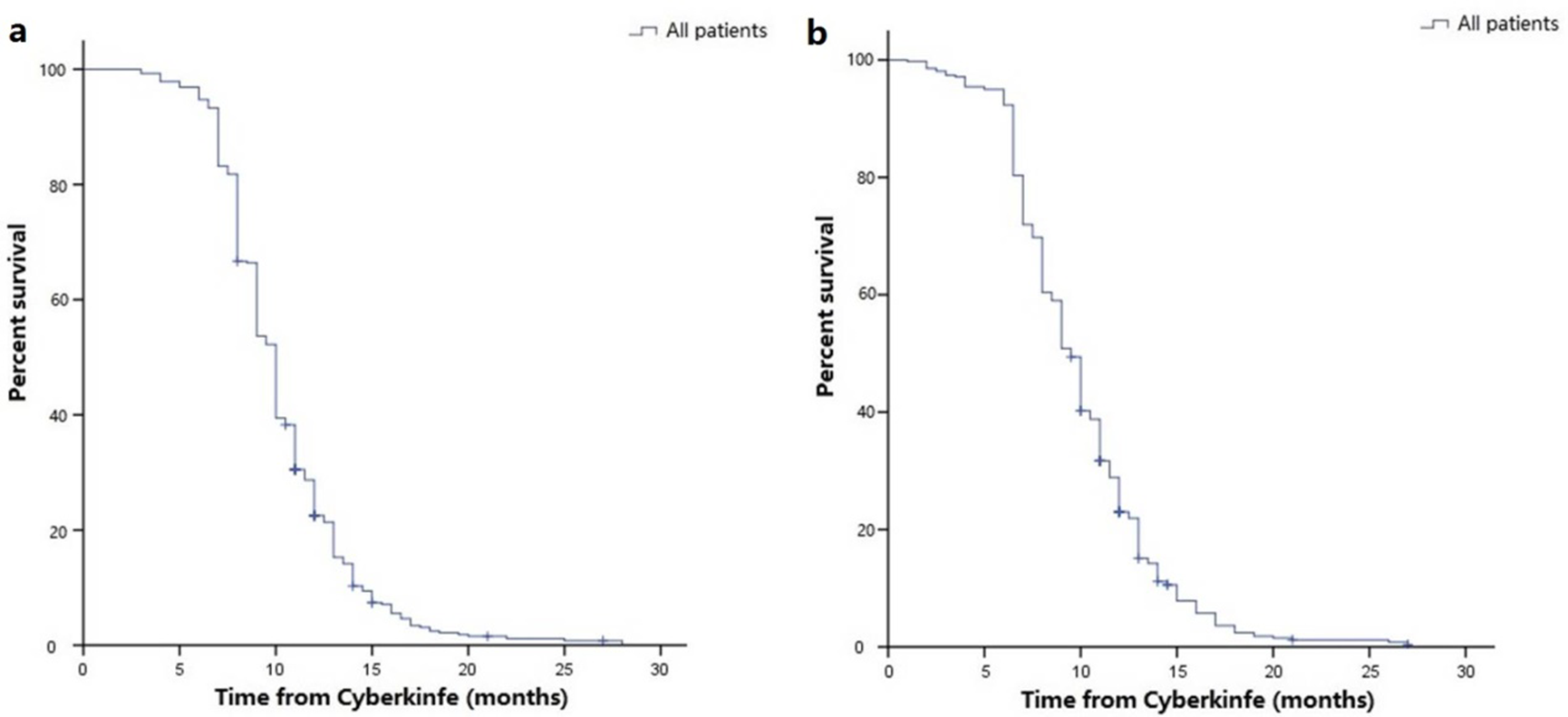

Supplement: Supplementary file 2 — Figure S2. LRFS (A) and DMFS (B) of all patients. [file CAM4-6-2263-s002.tif]
