# Supplementary material for: Prognostic role of stereotactic body radiation therapy for elderly patients with advanced and medically inoperable pancreatic cancer
Source: Cancer Med. 2017 Aug 23;6(10):2263–70. doi: 10.1002/cam4.1164 (PMC5633558; doi:10.1002/cam4.1164)
Supplement: Supplementary file 3 — Table S1. Factors associated with OS. Table S2. OS and PFS of patients at different stages, different responses, and different changes in CA19‐9 levels. Table S3. Factors associated with PFS. Table S4. Factors associated with LRFS. Table S5. LRFS and DMFS of patients with different stages, different responses, and different changes in CA19‐9. Table S6. Factors associated with DMFS. [file CAM4-6-2263-s003.docx]

Table 1 Factors associated with OS

| Factors | Univariate analysis | | | Multivariate analysis | | |
| --- | --- | --- | --- | --- | --- | --- |
|  | HR | 95% CI | *P* value | HR | 95% CI | *P* value |
| Age | 1.00 | 0.98-1.02 | 0.95 |  |  |  |
| Tumor diameter | 1.29 | 1.19-1.40 | <0.001 |  |  |  |
| Baseline CA19-9 | 1.02 | 0.97-1.05 | 0.13 |  |  |  |
| Stage | 2.72 | 2.30-3.22 | <0.001 | 2.38 | 1.98-2.87 | <0.001 |
| Prescription dose | 0.98 | 0.95-1.00 | 0.10 |  |  |  |
| BED_10_ | 0.99 | 0.97-1.00 | 0.05 |  |  |  |
| Tumor response | 0.20 | 0.16-0.24 | <0.001 | 0.20 | 0.16-0.25 | <0.001 |
| Normalization of CA19-9 | 0.57 | 0.50-0.66 | 0.001 | 0.76 | 0.65-0.88 | <0.001 |

Table 2 OS and PFS of patients at different stages, different responses, and different changes in CA19-9 levels.

| Patients | OS | | PFS | |
| --- | --- | --- | --- | --- |
| Stages | Median (m) | 95% CI (m) | Median (m) | 95% CI (m) |
| Borderline resectable | 13.0 | 12.5-13.5 | 11.5 | 10.9-12.1 |
| Locally advanced | 10.0 | 9.6-10.3 | 8.0 | 7.7-8.3 |
| Metastatic | 8.0 | 7.8-8.2 | 6.5 | 6.4-6.6 |
| Tumor response |  |  |  |  |
| CR | 18.0 | 16.0-19.9 | 17.5 | 15.0-19.9 |
| PR | 13.0 | 12.5-13.5 | 12.0 | 11.5-12.5 |
| SD | 10.0 | 9.7-10.2 | 8.0 | 7.8-8.2 |
| PD | 7.0 | 6.0-7.9 | 5.0 | 4.4-5.6 |
| Changes of CA19-9 |  |  |  |  |
| Normalization | 13.0 | 12.3-13.6 | 11.5 | 10.8-12.2 |
| Remain normal | 11.0 | 10.2-11.8 | 9.5 | 8.2-10.8 |
| Remain abnormal | 10.0 | 9.7-10.3 | 8.0 | 7.7-8.3 |

Table 3 Factors associated with PFS

| Factors | Univariate analysis | | | Multivariate analysis | | |
| --- | --- | --- | --- | --- | --- | --- |
|  | HR | 95% CI | *P* value | HR | 95% CI | *P* value |
| Age | 1.00 | 0.99-1.02 | 0.79 |  |  |  |
| Tumor diameter | 1.33 | 1.22-1.42 | <0.001 |  |  |  |
| Baseline CA19-9 | 1.01 | 0.98-1.03 | 0.09 |  |  |  |
| Stage | 2.97 | 2.50-3.53 | <0.001 | 3.06 | 2.53-3.72 | <0.001 |
| Prescription dose | 0.97 | 0.95-1.02 | 0.10 |  |  |  |
| BED_10_ | 0.99 | 0.97-1.01 | 0.05 |  |  |  |
| Tumor response | 0.11 | 0.09-0.15 | <0.001 | 0.09 | 0.07-0.12 | <0.001 |
| Normalization of CA19-9 | 0.56 | 0.49-0.64 | <0.001 | 0.72 | 0.62-0.83 | <0.001 |

Table 4 Factors associated with LRFS

| Factors | Univariate analysis | | | Multivariate analysis | | |
| --- | --- | --- | --- | --- | --- | --- |
|  | HR | 95% CI | *P* value | HR | 95% CI | *P* value |
| Age | 1.00 | 0.99-1.02 | 0.68 |  |  |  |
| Tumor diameter | 1.27 | 1.17-1.38 | <0.001 |  |  |  |
| Baseline CA19-9 | 1.01 | 0.98-1.03 | 0.15 |  |  |  |
| Stage | 2.27 | 1.94-2.66 | <0.001 | 1.81 | 1.52-2.15 | <0.001 |
| Prescription dose | 0.98 | 0.96-1.01 | 0.25 |  |  |  |
| BED_10_ | 0.99 | 0.98-1.00 | 0.14 |  |  |  |
| Tumor response | 0.21 | 0.18-0.26 | <0.001 | 0.23 | 0.19-0.28 | <0.001 |
| Normalization of CA19-9 | 0.58 | 0.50-0.66 | <0.001 | 0.76 | 0.66-0.89 | <0.001 |

Table 5 LRFS and DMFS of patients with different stages, different responses and different changes of CA19-9

| Patients | LRFS | | DMFS | |
| --- | --- | --- | --- | --- |
| Stages | Median (m) | 95% CI (m) | Median (m) | 95% CI (m) |
| Borderline resectable | 12.0 | 11.4-12.6 | 13.0 | 12.5-13.5 |
| Locally advanced | 10.0 | 9.6-10.4 | 10.0 | 9.6-10.4 |
| Metastatic | 8.0 | 7.9-8.1 | 6.5 | 6.4-6.6 |
| Tumor response |  |  |  |  |
| CR | 16.5 | 15.0-18.0 | 18.0 | 16.0-20.0 |
| PR | 13.0 | 12.7-13.3 | 13.0 | 12.5-13.5 |
| SD | 9.0 | 8.8-9.2 | 9.0 | 8.7-9.3 |
| PD | 6.0 | 5.4-6.6 | 5.0 | 4.2-5.7 |
| Changes of CA19-9 |  |  |  |  |
| Normalization | 12.0 | 11.4-12.6 | 13.0 | 12.2-13.8 |
| Remain normal | 11.0 | 10.1-11.9 | 10.0 | 9.3-10.7 |
| Remain abnormal | 9.0 | 8.8-9.2 | 8.5 | 8.1-8.9 |

Table 6 Factors associated with DMFS

| Factors | Univariate analysis | | | Multivariate analysis | | |
| --- | --- | --- | --- | --- | --- | --- |
|  | HR | 95% CI | *P* value | HR | 95% CI | *P* value |
| Age | 0.99 | 0.98-1.01 | 0.89 |  |  |  |
| Tumor diameter | 1.34 | 1.23-1.45 | <0.001 |  |  |  |
| Baseline CA19-9 | 1.00 | 0.99-1.01 | 0.11 |  |  |  |
| Stage | 3.32 | 2.76-3.98 | <0.001 | 3.39 | 2.75-4.20 | <0.001 |
| Prescription dose | 0.97 | 0.95-1.00 | 0.58 |  |  |  |
| BED_10_ | 0.98 | 0.97-0.99 | 0.03 |  |  |  |
| Tumor response | 0.17 | 0.14-0.21 | <0.001 | 0.16 | 0.13-0.20 | <0.001 |
| Normalization of CA19-9 | 0.56 | 0.49-0.64 | <0.001 | 0.77 | 0.67-0.89 | <0.001 |
